# Total gastrectomy for the treatment of Menetrier’s disease persistent to medical therapy: A case report

**DOI:** 10.1016/j.ijscr.2020.06.033

**Published:** 2020-06-30

**Authors:** Christos Parianos, Chrysanthi Aggeli, Antigoni Sourla, Georgios Nikolaos Zografos

**Affiliations:** a 3rd Department of Surgery, General Hospital Of Athens ‘G.Gennimatas”, Greece; b Pathology Department Medical School, University of Athens, Athens, Greece

**Keywords:** Menetrier’s disease, Total gastrectomy, Thrombophilia, EGFR receptor’s antibody, Case report

## Abstract

**Background:**

Menetrier’s disease is a rare hyperproliferative protein-losing gastropathy of the gastric foveolar epithelium. It is characterized by giant hypertrophic folds, excess mucus secretion, decreased acid secretion and hypoproteinemia due to selective loss of serum proteins across the gastric mucosa. The discovery of transforming growth factor-α overexpression opened the way of epidermal growth factor receptor blockade with cetuximab as first-line treatment modality for Menetrier’s disease.

**Case report- ethods:**

We present the case of a 46-year-old female patient with Menetrier’s disease. The diagnosis was based on clinical, endoscopic and histological criteria. Two years before the diagnosis of the disease the patient had an episode of deep vein thrombosis and pulmonary embolism. Real time PCR revealed mutation of the gene responsible for coagulation factor II. The patient received anticoagulation therapy and after a period of 6 months a severe anemia due to a gastrointestinal bleeding was presented. The appropriate investigation revealed the presence of Menetrier’s disease. The patient was referred to the surgical clinic with persistence of symptoms such as excessive weight loss, anemia and weakness, even after multiple medical treatment, including the monoclonal antibody against the EGFR receptor. A total gastrectomy was performed and the postoperative course was uneventful. One year follow up showed remarkable improvement of her health status.

**Conclusion:**

A combination of clinical, laboratory, endoscopic and histopathologic findings is necessary for the diagnosis of this rare disease. Gastrectomy is the treatment of choice for those patients with intractable symptoms and signs refractory to medical therapy.

## Highlights

• The etiology of Menetrier’s disease is still unclear and no definite diagnostic criteria exist.
• Our patient presented with a lower extremity DVT as initial sign of Menetrier’s disease.
• Overexpression of transforming growth factor-α (TGF- α) seems to play a significant role in the pathogenesis of the disease and erbitux is one of the main therapeutic options.

## 1 Introduction

Menetrier’s disease is a rare acquired condition of the stomach that is characterized by giant mucosal folds in the gastric fundus and body, diminished acid secretory capacity and a protein losing state with hypoalbuminemia [1]. It is also called protein losing hypertrophic gastropathy. It was first described by the French pathologist Pierre Menetrier in 1888 [2]. To date fewer than 1000 total cases have been reported [3].

Most common symptoms include epigastric pain with fullness, nausea or vomiting and a generalized peripheral edema with hypoalbuminemia[1] Gastrointestinal bleeding and diarrhea have also been described [4]. Basal and stimulated gastric acid secretion is usually low or normal and serum gastrin levels may show small to moderate elevation [1]. The most frequently observed laboratory findings are hypoalbuminemia, hypochlorhydria, elevated serum gastrin and iron deficiency anemia [5]. Radiologically, the wall of the gastric body and fundus is diffusely thickened, often with antral sparing. Endoscopy reveals giant rugal edematous gastric folds symmetrically enlarged or seldom asymmetrically enlarged with polypoid appearance [6]. A full thickness biopsy is required to reveal the loss of the deep glandular component [7]. Histology shows diffuse foveolar hyperplasia with cystic dilatation of the glandular portion of the gastric mucosa and the absence of significant inflammatory infiltrate [8]. The giant mucosal hypertrophy and hyperplasia consists mainly of gastric mucus cells while parietal and chief cells are notably diminished in number [8]. Menetrier’s disease has also a recognized premalignant potential although the precise risk of progression to gastric cancer is not known [9,10].

First-line treatment usually consists of a high-protein diet, proton pump inhibitors, eradication of helicobacter pylori or CMV and octreotide long-acting release [11]. Several studies reported regression of disease after treatment with the monoclonal antibody against the EGFR receptor [9]. But the only satisfactory treatment has historically been and remains the surgical intervention with total or partial gastrectomy [4].

## 2 Patient and methods

A 46-year-old female patient was admitted for surgical treatment after one year from the diagnosis of Menetrier’s disease because of the persistence of her symptoms. She had no relevant family or surgical history.

From her personal history, the patient had an episode of deep vein thrombosis at the right saphenofemoral junction and pulmonary embolism two years before. After that episode the patient followed a laboratory workup which revealed normal levels of protein S, protein C and antithrombin III. Real time PCR revealed mutation of the gene responsible for coagulation factor II (gene G20210A) Heterozygote, and normal genotype for the gene responsible for coagulation factor V (Gene V Leiden- G1691A). An anticoagulation therapy was initiated and received for six months.

**Fig. 1:**
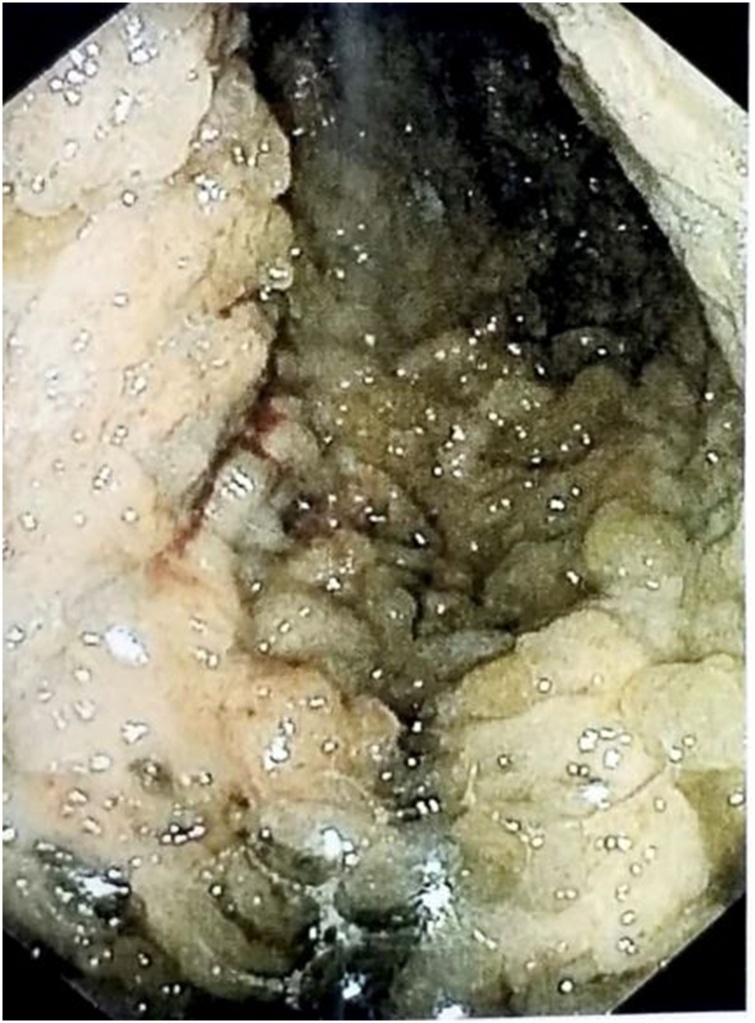
Preoperative upper endoscopy.

**Fig. 2:**
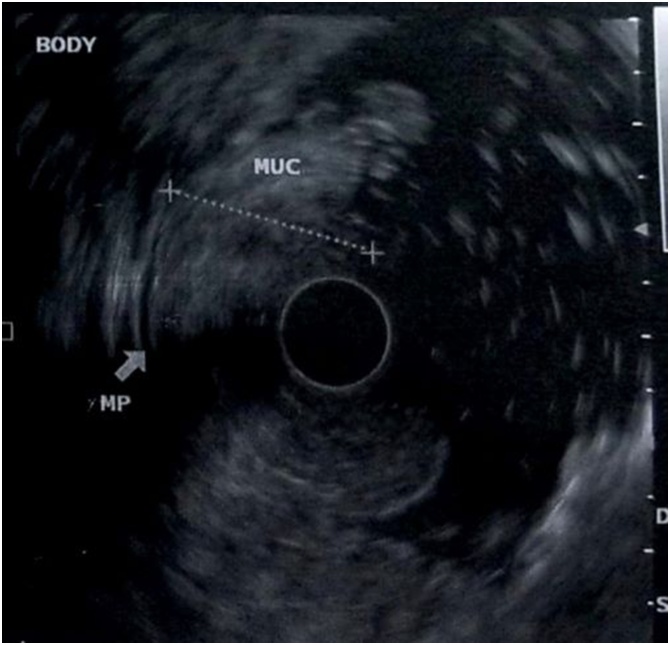
The endoscopic ultrasound shows the severe mucosal thickness.

**Fig. 3:**
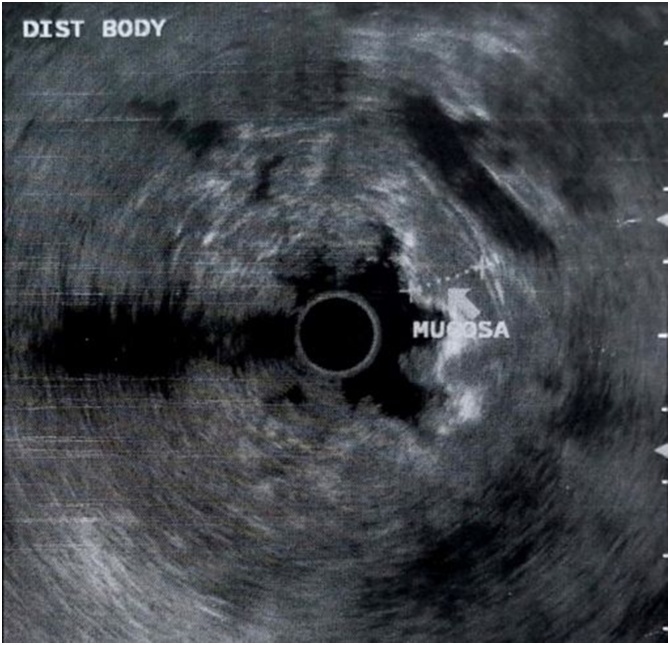
Endoscopic ultrasound from the distal body of the stomach.

**Fig. 4:**
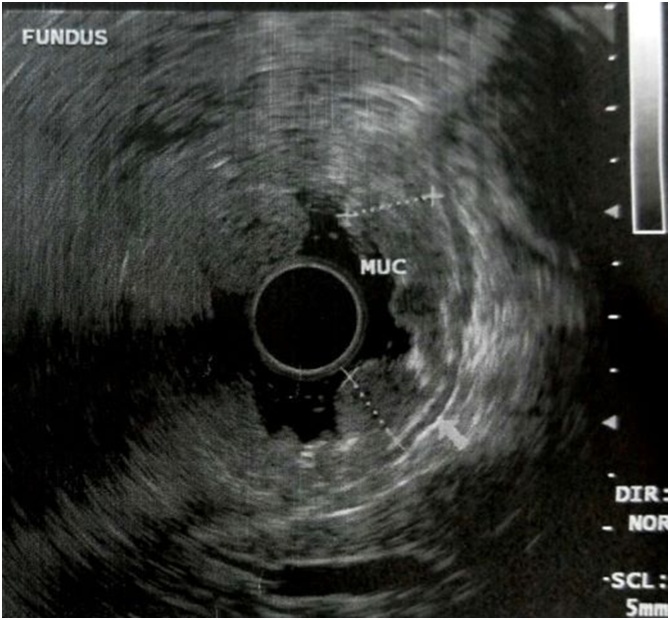
Endoscopic ultrasound from the fundus of the stomach.

After that period the patient developed anemia, she was admitted to the emergency room with hematocrit 16 %. An upper and lower gastrointestinal endoscopy was performed. Upper endoscopy revealed large rugal folds in the body and fundus of the stomach and reddish mucosal stimulations in the antrum (figure 1). There was no evidence of Helicobacter pylori or cytomegalovirus (CMV) immunohistochemical staining. The diagnosis of Menetrier’s disease was the first option in association with the clinical and laboratory picture. With no sign of lymphohyperplastic disease. Colonoscopy showed the presence of several small hyperplastic polyps which were resected with polypectomy bronchus. An abdominal computed tomography (CT) scan showed her stomach to be significantly enlarged and thickened. The patient received treatment with Erbitux for six months. The symptoms persisted and the patient presented also severe anemia and folliculitis in the face. An upper endoscopy showed no improvement in the lesions of the stomach despite the medical therapy. An endoscopic ultrasound (EUS) confirmed the severe thickness-hypertrophy of the mucosal, especially in the region of the fundus and body (8.5 mm in the fundus, 2 cm in the body) with normal imaging of the submucosal and muscle tissue in the same parts of the stomach(figures 2-4). Antrum and esophagus were normal on EUS imaging and no pathological regional lymph nodes were noted. A surgical treatment was proposed.

**Fig. 5:**
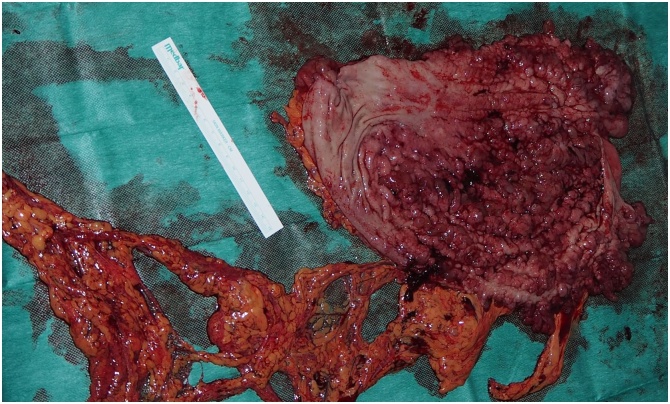
Specimen of the gastrectomy.

**Fig. 6:**
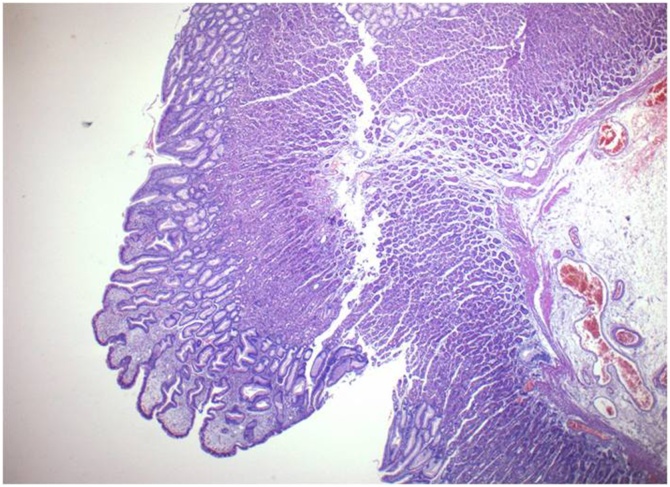
Elongated and hyperplastic foveolae (magnif. x4).

**Fig. 7:**
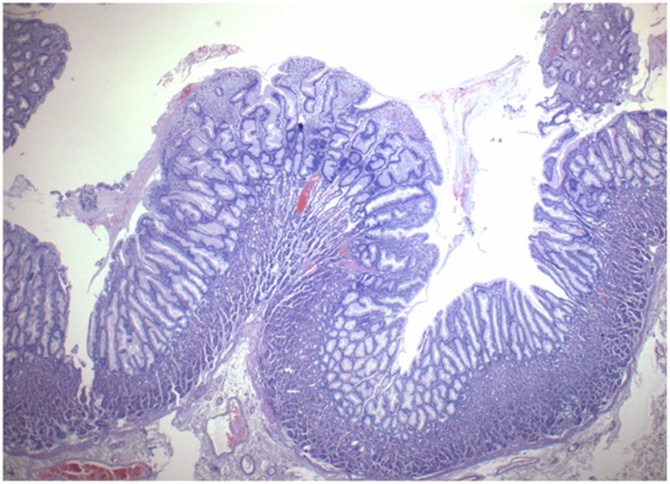
Tortuous and dilated hyperplastic gastric pits (magnif. x4).

**Fig. 8:**
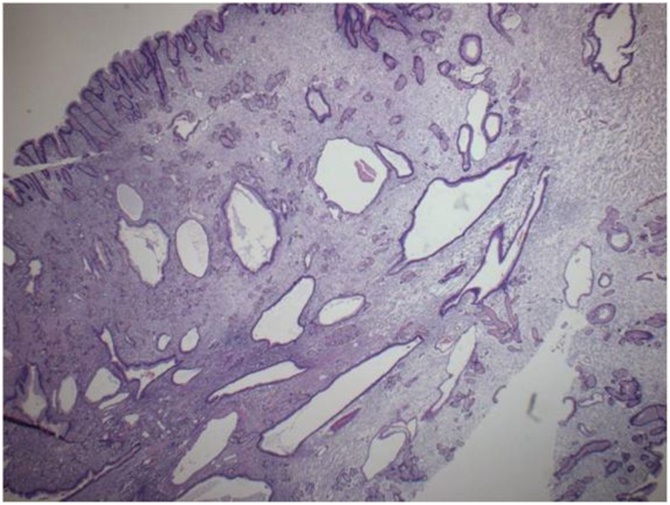
Glandular atrophy and cystic dilatation of glands wich extended to the submucosa (magnif. x10).

**Fig. 9:**
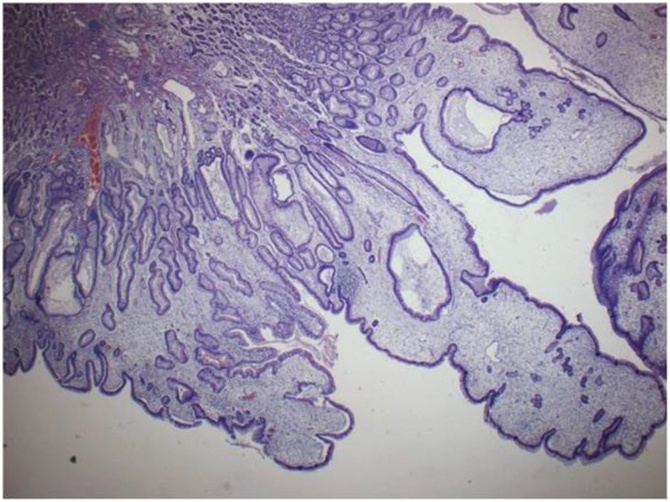
Significant edema and chronic inflammatory infiltration of the lamina propria (magnif. x10).

On physical examination on her present admission, no significant findings were revealed, except for excessive weight loss with weakness and skin rash on her face, a side effect from the medical treatment. The patient was cachetic with a flat abdomen. Laboratory investigations showed hypoalbuminemia (total protein: 5.6 g/dl, albumin: 2.6 g/dl) and a low serum iron level and hematocrit (iron 12 μg/dl, hct:33.1 %) (Fig. 1, Fig. 2, Fig. 3, Fig. 4, Fig. 5, Fig. 6, Fig. 7, Fig. 8, Fig. 9).

## 3 Surgical procedure –results

A midline incision was made. The size of the stomach was extremely large a proportion to the patient’s body type. The wall of the stomach was extremely thick through all its extent. A total gastrectomy was performed with the use of staplers and energy device (Fig. 5). An end-to-side stapled esophagojejunostomy was created with the use of circular anastomotic stapler diameter 21 mm and a hand sewn side to side anastomosis between the proximal (biliopancreatic limb) jejunum and the Roux-en-Y limb was also performed. Two surgical drainages were placed, one at the duodenal stump and the other at the esophagus jejunum junction.

The patient had a normal post- operative course. The 10th postoperative day an esophagram was obtained in the radiography department. No leakage was identified and a liquid diet was initiated and then increased gradually according to her tolerance.

The pathology report confirmed the endoscopic diagnosis of Menetrier’s disease (Fig. 6, Fig. 7, Fig. 8, Fig. 9). There were exaggerated gastric folds which involved mainly the body and the fundus of the stomach with foveolar hyperplasia. There was edema and capillary congestion of the chorion with elements of chronic unicellular inflammation. Several hyperplastic polyps in the antrum were noted with no signs of dysplasia or cancer. Thirty regional lymph were reactive due to the inflammation.

One year after the operation the patient is free of symptoms and has regained much of her weight. The levels of albumin and hematocrit are in the normal range. She follows a prophylactic anticoagulation therapy.

## 4 Discussion

The etiology of Menetrier’s disease is still unclear and no definite diagnostic criteria exist. A close association of clinical, laboratory, endoscopic and histopathologic findings is required to reach the final diagnosis. Primarily observed in male adults, it presents symptoms such as epigastric pain, vomiting, anorexia, anemia, weight loss and peripheral edema due to protein loss across the gastric mucosa [12,13]. In normal gastric mucosa, the pit to gland ratio is 1:4, but in Menetrier’s disease this ratio is usually reversed as the surface mucous cells compartment expands to occupy nearly the entire mucosal thickness [14]. Overexpression of transforming growth factor-α (TGF- α) seems to play a significant role in the pathogenesis of the disease [4]. TGF-a is a ligand that binds and activates EGFR which in turn results in proliferation of epithelial cells of the mucous cell compartment and inhibits gastric acid secretion [15,16]. Both EGFR and TGF-α are expressed in the normal gastric mucosa. The overexpression of them is related to Menetrier’s disease [17].

Gastric infection with CMV, a frequent cause of Menetrier’s disease in children, has also been associated with EGFR as the virus provoke overexpression of TGF-a and cell proliferation [18].

Menetrer’s disease is also strongly associated with helicobacter pylori (HP) infection though there are cases negative for the infection as it was our patient [19]. HP is considered to be the offending agent in antral gastritis and duodenal ulcer disease [20]. Its role in Menetrier’s disease is less clear. Some support that the gastric mucosa in the disease may be more susceptible to infection [20]. Several studies though have reported that the eradication of the helicobacter pylori succeeds complete clinical and morphologic recovery [21,22].

A relationship between Menetrier’s disease and cancer presentation has been observed in approximately 10 % of the cases probably because of the hypertrophic changes of the gastric mucosa and the consequent increased cell proliferation [23]. It may concern gastric carcinoma or gastric lymphoma but the association of the disease with cancer has yet to be confirmed due to its rarity [12]. It is anyway safe to monitor these lesions with regular endoscopic examinations and biopsies [12]. In the present case, the patient received a surveillance endoscopy 1 to –2 times per year. There was no indication of gastric carcinoma in anyone of the endoscopic interventions but the patient suffered from the persistence of the symptoms and she was also concerned about the potential development of cancer.

Another issue related with the disease is thrombophilia. There are reported cases of unprovoked venous thrombosis associated with protein losing diseases such as gastropathies, nephrotic syndrome and inflammatory bowel diseases [24,25]. In all these cases there was a significant protein losing state concomitant with thrombophilia that was derived from a defect in one or more clotting factors [24]. Our patient presented with a lower extremity DVT as initial sign of Menetrier’s disease. Gastrointestinal bleeding begun only after the initiation of anticoagulation therapy. The laboratory work up revealed mutation of the gene responsible for coagulation factor II. Menetrier’s disease was discovered in the investigation for the gastrointestinal bleeding.

A variety of diseases are associated with enlarged mucosal folds in the stomach rendering the diagnosis of Menetrier’s disease sometimes challenging. Zollinger-Ellison syndrome, malignancies, various gastridities, upper gastrointestinal polyps and massive gastric polyposis [26,27].

The natural history of this disease can be self-limited and results in complete resolution, especially in children younger than 10 years old. In adult patients it is a progressive disorder and symptoms may persist for years [28]. There are no reports of spontaneous regression of the disease in patients who have symptoms for more than 6 months [9].

There is no evidence-based guideline for the treatment of Menetrier’s disease. The first and main treatment is the nutritional support with high protein intake diets. Multiple medical treatments have been utilized but they don’t seem to provide consistent benefit and control trials are lacking [29]. Eradication of helicobacter pylori and CMV in case of their detection is necessary and sometimes effective. Ganciclovir may be effective in CMV related Menetrier’s disease in children [30]. Proton pump inhibitors or Histamine-2 receptor antagonists are usually given. The use of somatostatin analogs as octreotide have been used as they act on the EGFR on the cell surface [31]. Erbitux (cetuximab) is a monoclonal antibody directed to the ectodomain of the EGFR that prevents ligand binding. It is administered intravenously. The most frequent side effects are folliculitis and diarrhea [32].

Surgical treatment, in terms of total or partial gastrectomy, is recommended for cases with persistent and debilitating symptoms or when there is concern for gastric cancer development. Despite Menetrier’s disease affects mainly the fundus and body of the stomach, a total gastrectomy is favoured over a partial gastrectomy because of an enhanced quality of life and reduced surgical complications [33]. Moreover it reduces the risk of future presentation of malignancy and saves the patient from the need of future endoscopic surveillance.

## 5 Conclusion

The diagnosis of Menetrier’s disease has been significantly refined in the last years and the confirmation of the disease is made by the combination of clinical and laboratory findings as also the endoscopy and the histology of the biopsy material. With the recent discovery of the EGFR inhibitor, cetuximab, useful because of TGFa overproduction in this disease, a new first-line therapy is available as the last effort to avoid the operation. But when the symptoms persist total or partial gastrectomy remains the last and effective treatment for cure from the disease.

## Conflicts of interest

The authors have no conflicts of interest to declare

## Sources of funding

There was no funding for this manuscript

## Ethical approval

The manuscript was exempted from ethical approval by the scientific council of our hospital as it is a simple presentation of a case report, with the written consent of the patient.

## Consent

Written informed consent was obtained from the patient for publication of this case report and accompanying images. A copy of the written consent is available for review by the Editor-in-Chief of this journal on request.

## Author contribution

1. Christos Parianos: acquisition, analysis and interpretation of data, drafting of manuscript, critical revision

2. Chrysanthi Aggeli: study conception and design, analysis and interpretation of data, critical revision

3. Antigoni Sourla: acquisition, analysis and interpretation of data, drafting of manuscript

4. Georgios N. Zografos: critical revision

## Contributors

1. Dimitrios Kypraios MD: He performed the endoscopic ultrasound and provided the related photos

2. Periklis Apostolopoulos MD: He performed the endoscopy and provided the related photos

3. Dimitra Angeli MD: She offered her support with the grammar and spelling of the manuscript

## Registration of research studies

• Name of the registry: Research Registry
• Unique identifying number or registration ID: researchregistry5732
• Hyperlink to your specific registration (must be publicly accessible and will be checked): www.researchregistry.com

## Guarantor

Chrysanthi Aggeli

Christos Parianos

## Provenance and peer review

Not commissioned, externally peer-reviewed
